# A Method of Making Rubber Plates without the Medium of Wax

**Published:** 1907-06

**Authors:** J. B. Bigham


### A METHOD OF MAKING RUBBER PLATES WITHOUT THE MEDIUM OF WAX.

By Dr. J. B. Bigham, D. D. S.

S. C. State Dental Association, 1906.

Prosthetic Dentistry is the subject assigned me and the special phase of that subject I wish to discuss is rubber plates, or rather the method of making rubber plates without the medium of wax.

The method referred to, and which I will describe in a few moments, I have used exclusively for the past six months or more and can truthfully say that by it I have obtained better results, have had more perfectly fitting plates, and a satisfying confidence before putting in plates that they will fit, than I have ever been able to have in the same length of time during the twenty-two years of my dental practice.

The first requirement to secure a good fitting plate by this method, as by all others, is to obtain a perfect cast of the mouth from a carefully taken impression. Having the model fairly dry and set in the articulator ready for setting up the teeth, I draw a line with some sharp pointed instrument around the ridge as high up as I think the rim of the plate should extend, having first examined the mouth to assist me in reaching my conclusions. This mark will be imprinted on the vulcanized plate and the rim can be trimmed accurately to it, and it is seldom that I have any more trimming to do. I next place my vacuum chamber metal, very thin, just enough to relieve the pressure over hard palate and then paint the whole cast over with what I will designate as rubber glue. A word in regard to this glue. I have found that rubber dissolved in chloroform alone inclines to peel off the model, but if a small per cent. of turpentine is used it gives it a fine adhesive quality, the chloroform being volatile, soon evaporates and we have an adhesive but comparatively dry surface to place the rubber upon. We are now ready to place the rubber on the model which is done very much in the same way that the wax is used in the old method. Any rubber can be used that is used by any other process. I use Doherty's No. 2, light colored, red rubber and perfection pink, made by the Consolidated Dental Manufacturing Company. I use these because I like their color and strength, and not because other rubbers will not work by this method as well. For an ordinary sized model I take about three-fourths of a sheet of rubber and warm it well either on the lid of a water vessel over the lamp used for heating water, or by holding it over the spirit lamp a sufficient distance to prevent burning. The size of the sheet, or more properly speaking, its length will be shortened by contraction in the heating process, so that three-fourths of a sheet will not often be too much, but if so, the surplus can be carefully trimmed and utilized in the further construction of the plate. Having the sheet of rubber well warmed, it is placed on the model, first well up in the deep arch and then gradually over the whole surface. When nearing the outer edge of rim the rubber can be trimmed with scissors or hot wax knife to conform to margin of rim indicated by mark made on model and referred to above. In placing rubber we will find that there will be a surplus in front caused by the conical shape of model and if none is cut out there will be an ugly crease, but by cutting out from top of ridge immediately in front of fraenum of lip a good sized V shaped piece, (the exact size to cut out will be learned by experience only), the rubber will fold over ridge smoothly and accurately without any creasing. To be sure the rubber is in contact with every part of model, it is firmly pressed down all over with the thumb or middle finger.

We are now ready for the pink rubber veneering which is warmed and placed just as far as we wish to use it, being careful to press it well against the red rubber to insure uniform and perfect adhesion. The point is now reached for the setting and arranging of the teeth which is done by holding them in the flame of the alcohol lamp until rather hot and then pressing them down to proper place and as each tooth is set, secure it more firmly by placing a small piece of rubber on inside of arch around pins extending to rubber base plate which holds it approximately in proper place until all the teeth are set. We now go around the outer edge of rim and add any additional rubber, either red or pink, to fill any inequalities or give additional thickness to rim of plate. Having done this and seeing that all the teeth are in their proper relations to each other and to the opposite teeth either natural or of like kind, I carefully invest the outer side of teeth and rubber allowing the investment to gently come over grinding or incisal edge of teeth sufficiently thick—say one-fourth to three-eighths of an inch—to give firmness and strength to investment. This investing is done while model and teeth are still on articulator. After the investment has hardened sufficiently, I take my wax knife and after warming it pack the rubber we have already in place well around inside neck and pins of teeth and then add whatever amount of rubber is necessary and force it in by heavy pressuse using thumb and fingers to accomplish this end. This last process is a very simple and easy one, and can be done in a few moments.

The rubber all being in place, I invest the palatal portion which completes the investment and entirely envelopes the case in plaster. The whole thing is removed from the articulator, trimmed enough to allow it to go into the flask and invested, vulcanized and finished in the usual way. The teeth can be set up and all rubber placed without the use of the investment I have spoken of, but the investing is a short and easy process and very much facilitates the placing of the rubber on the inside of the teeth. The only objection to the investment that can be raised is that it so enlarges the case that a good deal of trimming is necessary to get it into an ordinary sized flask, especially if the plate be an extra large one. This objection can easily be obviated by getting extra large flasks, which are well to have in all offices anyway to meet exceptionally large cases. Aside from the fact that we obtain a better fitting set of teeth, the reason for which I wish to speak presently, we shorten the process very materially and obviate the necessity of soiling up our hands with waxy, messy flasks of which we are all too familiar.

In repair work this method shortens the process by one-half and by it we secure highly satisfactory results and if any of the brethren do not use this new method in any form it would be well for them to try it to the extent at least in repairing, and I am confident they would be well pleased. The principal reason we secure better fitting plates by this method is that no force is used in placing the rubber and there is no possibility of a change in shape of model, whereas by the old method, the model is boiled in water, which no doubt, to some extent, softens it and when in this condition with a surplus of rubber in flask it is screwed up using considerable force in doing so, and I think in many instances the little delicate ruga and prominences are dented and flattened, and for this reason, if we have no greater mutilation of model, we do not secure that perfect adaptation that we are able to secure where no such pressure has been used, and where no change of model is a possibility. I do not claim originality for this method, excepting in some of the details. The kind of glue I use is of my own formula, the investment to facilitate the packing of rubber on inside of teeth, is my own idea, and I consider it a valuable one and the exclusive use of rubber instead of vulcanizable gutta percha or partly rubber and partly vulcanizable gutta percha, is of my own adoption.

The question may be asked why I use rubber in preference to vulcanizable gutta percha, and I will answer by saying that rubber has been weighed in the balances and found not wanting, having every quality in the finished plate that we could possibly hope for in vulcanizable gutta percha, and I am inclined to think it superior, so far as my experience goes, to gutta percha.

There is one quality that the gutta percha preparation has that may make it more desirable than rubber for the use of some dentists and that is it can be warmed and shaped to the model and when cool it is comparatively firm and retains its shape so that after the teeth are set up, they can be tried in the mouth. This is a practice that some dentists claim to invariably follow, but I have found such a course impracticable, so this does not deter me from the use of the rubber, which on account of its pliability in its unvulcanized state, cannot be used in this way and must be pasted down to the model at once.

I am no word painter, but I trust by the use of plain language, I have made myself fairly well understood, and if any one, is interested and desires a fuller detail in regard to any part of the process, I shall be glad to answer any questions.

